# A24 AN ULCERATIVE COLITIS-ISOLATED PATHOBIONT CAN DEGRADE MUCUS PRODUCED BY UC PATIENT-DERIVED COLONOIDS

**DOI:** 10.1093/jcag/gwad061.024

**Published:** 2024-02-14

**Authors:** A Gilliland, Y Chen, D Tertigas, M Surette, B Bressler, B Vallance

**Affiliations:** The University of British Columbia, Vancouver, BC, Canada; The University of British Columbia, Vancouver, BC, Canada; Biochemistry and Biomedical Sciences, McMaster University Faculty of Health Sciences, Hamilton, ON, Canada; Biochemistry and Biomedical Sciences, McMaster University Faculty of Health Sciences, Hamilton, ON, Canada; The University of British Columbia, Vancouver, BC, Canada; The University of British Columbia, Vancouver, BC, Canada

## Abstract

**Background:**

Inflammatory bowel disease (IBD) pathobionts are commensal microbes with pathogenic potential that may cause or exacerbate IBD symptoms. Some pathobionts (ex. *Escherichia coli*) reside at low levels in the lumen of a healthy gut but can rapidly grow in the inflamed colons of ulcerative colitis (UC) patients. To promote disease, these pathobionts must cross the colonic mucus barrier (comprised of MUC2) that separates the epithelium from luminal microbes. It is currently unclear how bacterial pathobionts cross the mucus barrier of UC patients.

**Aims:**

Using healthy and UC patient biopsy-derived colonic organoids (colonoids) and an air liquid interface (ALI) monolayer model, we investigated how the UC-isolated *E. coli* pathobiont p19A crosses the mucus barrier.

**Methods:**

Apical out healthy and UC patient biopsy-derived colonoids were infected with p19A to confirm this pathobiont exerts direct cytopathic effects on human colonocytes. Sequencing p19A’s genome, we found it contains two mucus degrading proteins (mucinases). Healthy human and UC colonoids, as well as mouse colonoids, were used to generate mucus-producing ALI monolayers. To detect p19A-mediated mucus degradation, concentrated p19A supernatant was incubated with ALI-derived mucus and degraded MUC2 proteins were detected by protein gel and MUC2 Western blot. MUC2 glycosylation was analyzed by protein gel and PAS staining. ALI monolayers were infected with p19A to evaluate mucus degradation and p19A localization by immunostaining.

**Results:**

The UC-isolated pathobiont p19A infected and exerted cytotoxic effects on apical out healthy and UC patient colonoids. By day 14, ALI monolayers were differentiated and covered by a thick mucus layer as assessed using brightfield microscopy. Mucus removed for mucinase assays was replenished within 7 days. p19A harbours proteins capable of degrading human ALI-, but not mouse ALI-derived mucus, *in vitro,* suggesting the presence of host-specific mucinases. Mucus produced by UC ALI monolayers showed reduced glycosylation and increased degradation both over time and by p19A proteins. Day 21 ALI-monolayers infected with p19A for 18 hours exhibited overt mucus degradation allowing p19A to infect the underlying epithelium.

**Conclusions:**

Patient-derived ALI monolayers produce a thick mucus layer that can be used to study pathobiont-mucus interactions. The UC pathobiont p19A disrupts apical out organoids and produces proteins that degrade ALI-derived mucus *in vitro,* with UC mucus being more susceptible to degradation than mucus from healthy controls. The results from our model suggest a potential mechanism for pathobiont-mediated mucosal barrier disruption in UC patients.

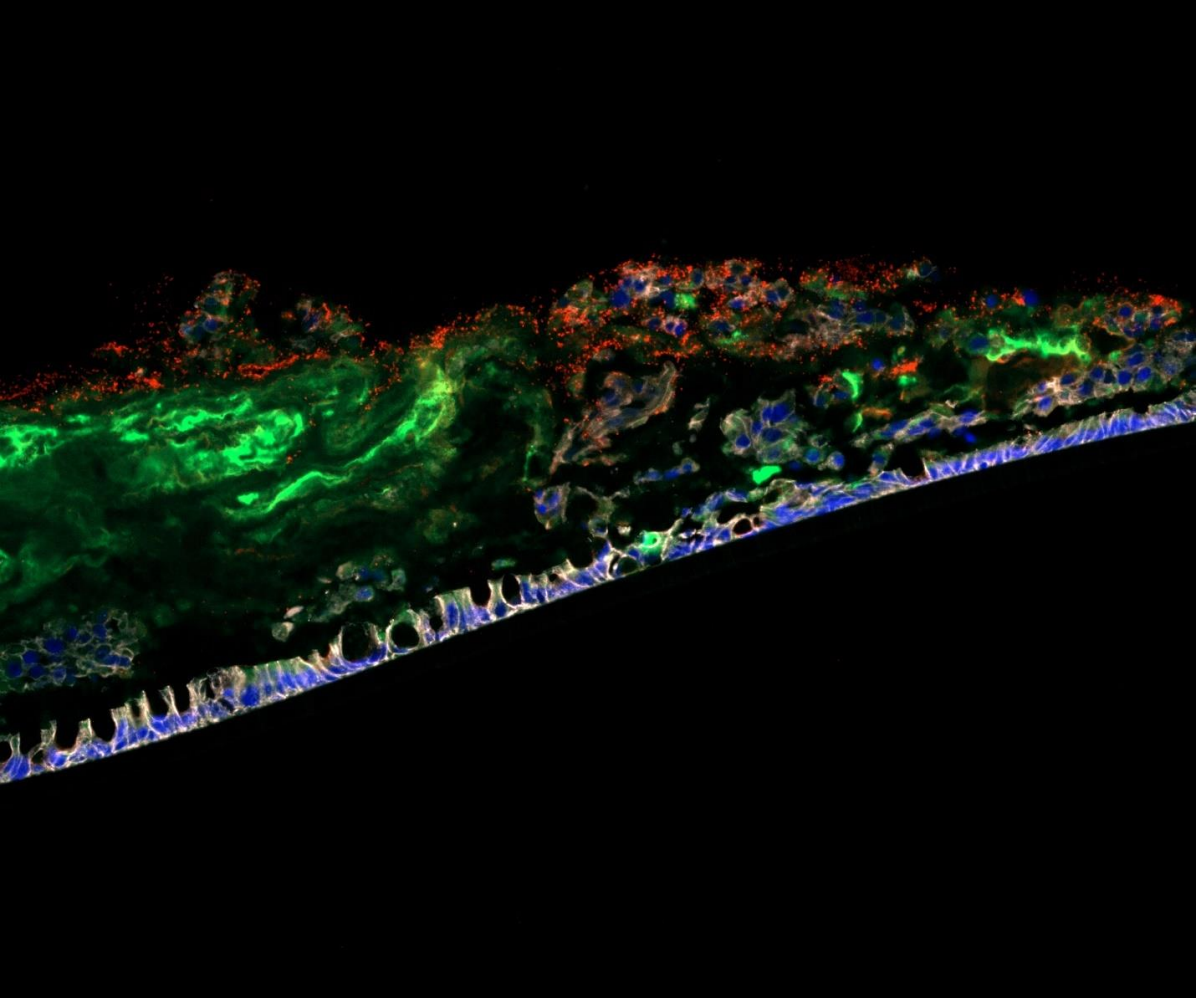

Patient-derived ALI monolayer (blue/white) produces a thick mucus layer (green) that can be degraded by the pathobiont p19A (red).

**Funding Agencies:**

CCC, CIHR

